# Study on the Degradation of the p–y Response of Offshore Wind Monopiles Under Vortex-Induced Loading

**DOI:** 10.3390/ma19214597

**Published:** 2026-10-30

**Authors:** Chenfeng Zong, Dongyang Ge, Yuhui Zhang, Tongchun Qin, Zhongyang Mao

**Affiliations:** 1 School of Urban Science and Offshore Engineering, Nantong Institute of Technology, Nantong 226001, China; 20246384@ntit.edu.cn (C.Z.); dongyang_ge_ntit@163.com (D.G.); 20243699@ntit.edu.cn (Y.Z.); qintongchun@163.com (T.Q.); 2 School of Materials Science and Engineering, Nanjing Tech University, Nanjing 210009, China

**Keywords:** offshore wind monopile, vortex-induced vibration, pile–soil interaction, cyclic lateral loading, p–y curve

## Abstract

Current-induced vortex shedding imposes repeated cross-flow loading on offshore wind monopiles, yet its effect on the lateral pile-soil response is not explicitly represented in conventional p-y formulations. This study used a validated three-dimensional ABAQUS model of a large diameter monopile in sand to evaluate static in-line drag, equivalent peak cross flow lift, and stabilized harmonic lift at representative elevations. Static and post-cyclic p-y curves were extracted after up to 10^4^ cycles and compared with the American Petroleum Institute (API) sand relation. The in-line load governed the short-term displacement and bending-moment response, whereas repeated cross flow loading progressively reduced the mobilized soil resistance. After 10^4^ cycles, the reduction reached approximately 6.0–12.5% at z = D/2 and 6.75–13.4% at z = D across the representative low and high load cases. The numerical curves also exhibited a lower initial soil reaction modulus than the API prediction within the investigated shallow zone. A modified p-y formulation was therefore developed by combining a nonlinear depth dependent reference modulus with a cycle dependent degradation coefficient. The formulation provides an engineering representation of VIV-related stiffness degradation for the investigated monopile sand system and loading range.

## 1. Introduction

Offshore wind turbines are increasingly deployed in deeper water and at larger rated capacities, which places greater demands on the stiffness, strength, and long-term serviceability of their supporting foundations. Monopiles remain a widely adopted solution because of their comparatively simple fabrication and installation, but they operate as dynamically sensitive soil–pile–tower systems under concurrent wind, wave, and current actions. Laboratory measurements have shown that currents can make a substantial contribution to the mean hydrodynamic load and introduce low-frequency fluctuations, while waves usually dominate the unsteady response when the two actions coexist [1]. For a cylindrical pile, alternating vortex shedding additionally generates a transverse lift force. Monopile experiments have directly measured the resulting vortex-induced vibration (VIV) and demonstrated that its amplitude and associated hydrodynamic damping can affect structural response and fatigue-related design quantities [2,3]. Consequently, the cross-flow component should not be judged solely by its peak magnitude: even when it is smaller than the in-line drag force, its repeated action may progressively alter the soil support mobilized around the embedded pile.

The lateral response of monopiles is commonly represented by the p–y method, in which the pile is treated as a beam supported by depth-dependent nonlinear springs. This framework is attractive for engineering analysis, but the conventional sand relations were calibrated mainly against small-diameter, relatively flexible piles and therefore do not fully reproduce the three-dimensional resistance mechanisms and rotation-dominated response of modern large-diameter monopiles. Recent numerical and physical studies have accordingly proposed revised static and cyclic p–y formulations [4], quantified the influence of prototype stress level on cyclic response [5], and developed lateral-capacity and load–rotation relations over broad ranges of pile geometry and sand state [6]. Centrifuge tests further indicate that relative pile–soil stiffness, rather than diameter alone, can govern the observed departure from the American Petroleum Institute (API) formulation [7]. Together, these findings show that direct use of the conventional curve can misrepresent both the initial soil-reaction modulus and the mobilized resistance of large-diameter monopiles.

Cyclic loading introduces an additional source of uncertainty because the soil response evolves with load history. Two-way centrifuge tests on dense sand have identified short-term changes in lateral resistance and base contribution [8], whereas model tests in saturated silty sand have reported either stiffening in loose material or degradation in denser material as the surrounding soil is densified or disturbed [9]. Numerical analyses with hypoplastic sand models likewise show that load amplitude and sequence can control pore-pressure accumulation, pile-head displacement, and ground settlement [10]. To translate such effects into practical design, cycle-dependent overlay formulations have been developed to transform a monotonic p–y curve according to the number of cycles and loading level [11]. More recent fully coupled analyses demonstrate that drainage assumptions can materially change the predicted response [12], while CPT-based and laboratory-calibrated models seek to reduce uncertainty in initial stiffness, transition-shape degradation, and accumulated deformation [13,14]. The direction and magnitude of cyclic evolution are therefore not universal; they depend on sand state, stress level, drainage, pile–soil relative stiffness, load ratio, displacement level, and number of cycles. Assessments of cyclic superposition approaches have shown that the treatment of successive load packages influences accumulated monopile rotation [15]. For asymmetric cyclic loading, p–y models have incorporated sand convection and modified reloading rules to represent different degrees of load reversal [16]. Two-dimensional formulations have further extended the p–y framework to multidirectional cyclic loading while accounting for sand collapse [17].

Despite this progress, current-induced VIV and cyclic pile–soil interaction are usually treated in separate parts of the analysis. Hydrodynamic studies resolve the oscillatory cross-flow force, whereas geotechnical studies commonly prescribe idealized cyclic loads that are not explicitly linked to vortex shedding along the exposed pile. In addition, recent centrifuge evidence continues to show substantial discrepancies between conventional sand p–y curves and the response of large-diameter monopiles, particularly in initial stiffness and shallow resistance [18]. A consistent engineering procedure is therefore needed to transfer current-dependent vortex-induced lift into the pile–soil model and quantify the post-cyclic p–y response. Building on existing cyclic p–y modelling approaches [4,11,19], this procedure should incorporate equivalent cross-flow loading at representative elevations while distinguishing the depth-dependent static stiffness correction from the progressive effect of repeated loading.

A consistent engineering procedure is therefore needed to transfer current-dependent vortex-induced lift into the pile–soil model, quantify the post-cyclic p–y response, and distinguish the depth-dependent static bias from the progressive effect of repeated cross-flow loading.

This study addresses that need using a validated three-dimensional finite-element model of a large-diameter monopile in sand. The current-induced load is decomposed into an in-line drag component and a cross-flow lift component; the transient peak lift is represented by an equivalent static load, and the stabilized vortex-shedding lift is applied as a harmonic cyclic load at representative elevations. Static and post-cyclic p–y curves are then extracted after up to 10^4^ cycles and compared with the API sand relation. The analyses show that the in-line load governs the short-term static deformation, whereas repeated cross-flow loading progressively reduces the mobilized lateral soil resistance. On this basis, a modified p–y formulation is developed by combining a nonlinear depth-dependent reference modulus with a cycle-dependent degradation coefficient. The formulation is intended as an engineering representation for the investigated monopile–sand system and loading range; broader application requires independent calibration against cyclic centrifuge or field data.

## 2. Numerical Model and Loading Procedure

An offshore wind monopile extends through the water column and is embedded in the seabed, so current loading applied to the exposed pile is ultimately resisted by bending of the steel tube and lateral reactions in the surrounding soil. The steady component of the current produces predominantly in-line drag, whereas alternating vortex shedding generates cross-flow lift that can act repeatedly during operation. For large-diameter monopiles, the resulting response depends not only on the external load but also on the stiffness and nonlinear resistance of the pile–soil system. A representative engineering model must therefore reproduce lateral load transfer in the embedded segment and apply the hydrodynamic actions at their corresponding elevations before the effect of vortex-induced loading on foundation performance can be assessed.

### 2.1. Finite-Element Model of the Monopile Soil System

**Figure 1:**
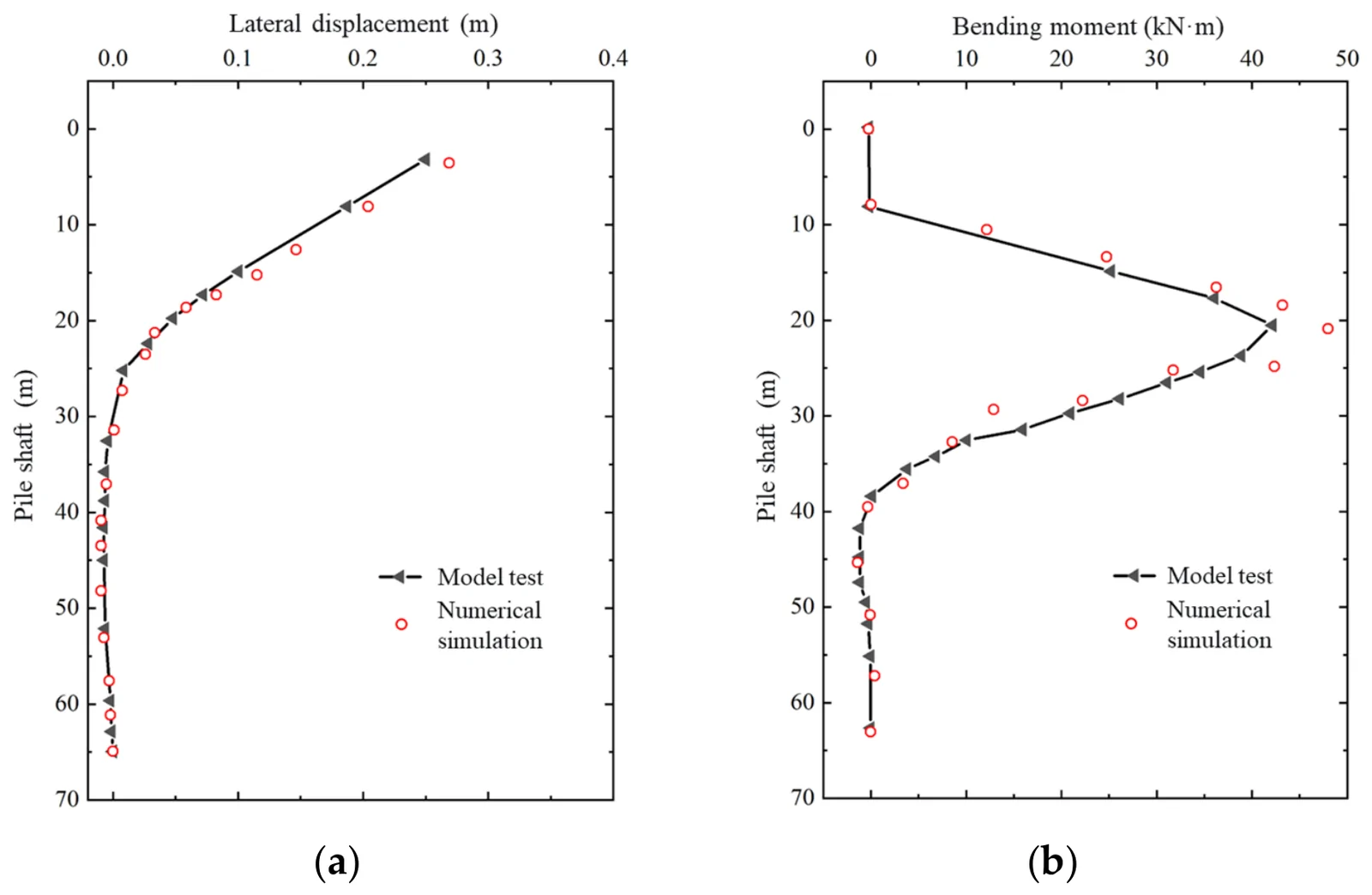
Comparison of measured and calculated pile responses in the centrifuge validation: (**a**) horizontal displacement and (**b**) bending moment.

A three-dimensional finite-element model was developed in ABAQUS to simulate the lateral response of the monopile–soil system. The modelling approach was first validated against the centrifuge model tests reported by Zhu et al. [20] for a large-diameter monopile subjected to lateral loading in dry Fujian standard sand. The pile was modelled as a linear-elastic hollow steel tube, and the sand was represented using the Mohr–Coulomb constitutive model. The parameters of the validation model are summarized in Table 1. Following the test procedure, a static lateral load of P = 4827 kN was applied at a height of 2.7D above the ground surface, where D is the pile outer diameter. The calculated distributions of lateral displacement and bending moment along the pile are compared with the centrifuge-test results in Figure 1a,b, respectively. With errors normalized by the maximum absolute experimental response, the normalized root-mean-square errors were approximately 3.8% for lateral displacement and 10.1% for bending moment; the corresponding maximum normalized discrepancies were approximately 9.6% and 23.1%. Although the monotonic centrifuge comparison does not directly validate cyclic degradation, the agreement in pile displacement and bending moment supports the adopted finite-element approach for representing pile–soil interaction and provides a basis for the subsequent cyclic analyses.

The validated approach was then applied to the engineering-scale monopile–soil model, whose geometry and material properties are given in Table 1. The monopile was modelled as a linear-elastic hollow steel pile embedded in sand governed by the Mohr–Coulomb constitutive model. The soil domain measured 80 m × 80 m × 80 m. Normal displacement was restrained on the four vertical boundaries, and all translational degrees of freedom were fixed at the base. The pile–soil interface was represented by hard contact in the normal direction and tangential frictional contact with a friction coefficient of 0.35. Both the pile and the soil were discretized using eight-node linear brick elements C3D8, with local mesh refinement around the pile to capture the pile–soil interaction.

The engineering model represents a cylindrical hollow steel monopile embedded in an idealized homogeneous fine-sand deposit. The soil parameters in Table 1 were selected with reference to the Engineering Geology Handbook [21] to define a consistent engineering parameter set rather than a site-specific calibration. The cohesion of 5 kPa is an assumed nonzero intercept in the adopted Mohr–Coulomb representation and should not be interpreted as measured intrinsic cohesion of clean sand. The elastic modulus of 56 MPa was used as a constant reference stiffness, without representing its dependence on stress level or strain amplitude. The dilation angle of 9°, lower than the friction angle of 39°, specifies the plastic volumetric expansion in the non-associated flow rule. The Mohr–Coulomb model is used as an idealized elastoplastic representation for comparing pile–soil responses; its fixed parameters do not explicitly describe cycle-dependent changes in sand stiffness or fabric.

**Figure 2:**
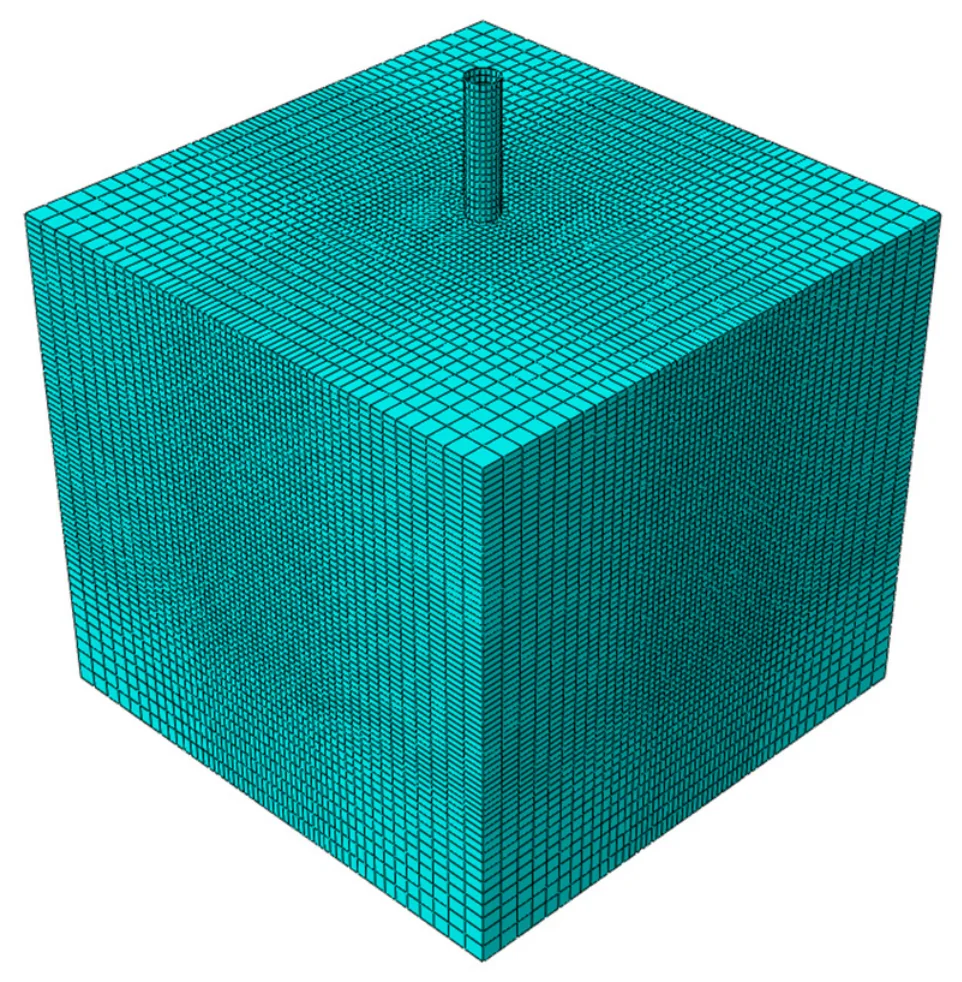
Three-dimensional finite-element mesh of the engineering monopile and surrounding soil domain.

The finite-element mesh of the engineering-scale monopile–soil model is shown in Figure 2. The model contains 168,690 eight-node linear brick elements (C3D8). A swept mesh was generated along the pile, while the surrounding soil was discretized using a graded mesh with a characteristic element size of 2 m near the outer boundaries and 0.6 m in the inner refined region around the pile. This refinement provides a finer spatial resolution for the local pile–soil response. The interface was represented using surface-to-surface contact, with hard contact in the normal direction and frictional contact in the tangential direction, with a friction coefficient of 0.35. The 80 m × 80 m × 80 m soil domain has a horizontal width of approximately 17.8D. Systematic mesh and domain sensitivity analyses were not performed, and numerical independence therefore remains unverified.

Before applying lateral loads, the initial stress state of the engineering model was established in a preliminary Static, General step in Abaqus. The analysis started from zero stress, without a predefined K_0_ stress field. Soil self-weight was introduced using a linear gravity amplitude increasing from 0 to 1 over the equilibrium step, while the boundary restraints specified above were maintained. The gravity turn-on calculation generated the initial stresses; a separate Geostatic step was not used.

Buoyancy in the saturated sand was represented using the submerged unit weight, γ′ = γ_sat_ − γ_w_ = 20.8 − 9.81 = 10.99 kN/m^3^. This value was used for the soil self-weight body force in the engineering model, with the saturated unit weight taken from Table 1. The C3D8 stress–displacement model did not solve a coupled pore-pressure field, so transient pore-pressure generation and dissipation were not simulated.

After gravity equilibration, the resulting stress state and full gravity load were retained for the lateral-loading analyses. The uncycled monotonic analysis and each independent cyclic-loading case began from this gravity-equilibrated state.

### 2.2. Equivalent Vortex-Induced Cross-Flow Loading and p–y Evaluation

The current-induced loads acting on the offshore wind monopile were resolved into in-line and cross-flow components. The cross-flow component was represented by the maximum lift force induced by vortex-induced vibration, whereas the in-line component accounted for the current-induced drag force. The cross-flow loads were determined from the vortex-induced lift forces calculated at different current velocities. Because the initial fluid–pile interaction produced a transient peak that was several times greater than the subsequent periodic lift amplitude, the maximum lift force was simplified as an equivalent static load to evaluate its effect on the monopile. The in-line static loads were calculated using the current-force formulation specified in the Code of Loads for Harbour Engineering (JTS 144-1-2017) [22]. The maximum static loads in the in-line and cross-flow directions are summarized in Table 2.

The preceding hydrodynamic simulations were conducted in Fluent using a two-dimensional slice model with the SST k–ω turbulence model and overset meshes. The cylinder diameter was 4.5 m, with a natural frequency of 0.1 Hz, a damping ratio of 0.005, and a mass ratio of 17. The water density and dynamic viscosity were 998 kg/m^3^ and 0.001003 Pa·s, respectively. Velocity-inlet, pressure-outlet, symmetry, and no-slip conditions were imposed at the upstream, downstream, transverse, and cylinder boundaries, respectively. The background and near-cylinder meshes contained 275,428 and 127,698 cells, with refinement within 2.5 cylinder diameters of the cylinder centre. The resulting Fluent simulation data have been compiled but have not been published separately.

For the equivalent load representation, the force magnitude is expressed as F = 0.5*ρ*Du^2^B, where *ρ* = 998 kg/m^3^, D = 4.5 m, u is the representative current velocity, and B combines the hydrodynamic coefficient with the effective loaded length. For Cases 1–5, the equivalent parameters for the transient peak lift are B_L,peak_ = (0.890670, 0.723670, 0.618521, 0.668003, 0.534402) m, and those for the in-line drag are B_D_ = (23.602761, 23.491427, 23.553279, 23.658428, 23.513694) m. Substituting these values into F_L,peak_ = 0.5*ρ*Du^2^B_L,peak_ and F_D_ = 0.5*ρ*Du^2^B_D_ gives the static loads listed in Table 2. For the stabilized cyclic lift, B_L,a_ = (0.668003, 0.450902, 0.450778, 0.400802, 0.350746) m, yielding the amplitudes in Table 3 through F_L_ = 0.5*ρ*Du^2^B_L,a_. The corresponding cyclic force is applied as F_L_(t) = F_L_cos(2πf_s_t), where f_s_ is the frequency listed in Table 3. Forces calculated using these SI units are obtained in N and converted to kN for presentation in the tables.

For the pile–soil analyses, the approximately periodic cross-flow lift was idealized as a prescribed, zero-mean harmonic force. For loading case i, the applied force was defined as:

(1) $$F_{i}(t)=F_{a,i}\cos(2\pi f_{i}t),F_{a,i}(t)=\frac{1}{2}\rho Du_{i}^{2}B_{L,a,i}$$

where F_a,i_ is the force amplitude, f_i_ is the prescribed cyclic frequency in Hz, and u_i_ is the representative current velocity. The equivalent parameter B_L,a,i_, with units of length, combines the effects of the lift coefficient and effective loaded length. It is defined from the adopted force amplitude as B_L,a,i_ = 2F_a,i_/*ρDu_i_*^2^.

The equivalent loading parameters, including the applied force amplitudes and frequencies representing the adopted periodic lift response, are listed in Table 3. Five representative elevations of 4, 8, 12, 16, and 20 m above the seabed were selected along the exposed pile segment, with corresponding current velocities of 1–5 m/s. Each case was applied independently as a concentrated cross-flow force at its specified elevation. The hydrodynamic loading was transferred to the pile–soil model in one direction, without updating the prescribed force in response to the subsequent pile–soil deformation. This representation evaluates individual combinations of load amplitude and application elevation.

The VIV-induced lift obtained from the preceding fluid calculations is represented by equivalent harmonic loading in the pile–soil model through one-way load transfer. The pile–soil analyses employed the built-in continuum stress–displacement and contact formulations in Abaqus. The normal pile–soil interaction was represented by hard contact, while tangential resistance followed the Coulomb friction law with a coefficient of 0.35. The gravity-equilibrated model described in Section 2.1 was used for the uncycled monotonic reference analysis and, independently, for each cyclic-loading case followed by monotonic loading at the mudline. The cyclic cross-flow force followed a cosine waveform using the case-specific amplitudes and frequencies in Table 3, with responses evaluated after 1, 10, 100, 1000, and 10,000 cycles. The cyclic analyses apply the harmonic cross-flow load without superimposing the mean in-line drag, which is considered separately in the static analyses. During the monotonic stages, the lateral load was increased incrementally, and the corresponding soil reactions and pile displacements were used to construct the p–y curves. The soil resistance p denotes the lateral soil reaction per unit pile length, and y denotes the corresponding lateral displacement of the pile at the selected depth below the seabed. The initial soil-reaction modulus K is defined as the initial tangent slope of the monotonic p–y loading branch. The uncycled static response is denoted by *N* = 0, while *N* = 1 denotes the response obtained through monotonic loading after one complete loading cycle.

Because the pile was modelled as linear elastic, structural failure was not simulated. The L/500 displacement criterion discussed in [23] is treated here as a serviceability reference, where L denotes the embedded pile length. For L = 53 m, this displacement equals 0.106 m. The reported load of 5419 kN corresponds to a mudline displacement of approximately 0.1 m and is therefore designated as a reference serviceability load, *H*_ref_, rather than an ultimate failure capacity. A maximum monotonic mudline load of *H*_max_ = 5.5 MN was applied incrementally to obtain the static and post-cyclic p–y curves.

**Figure 3:**
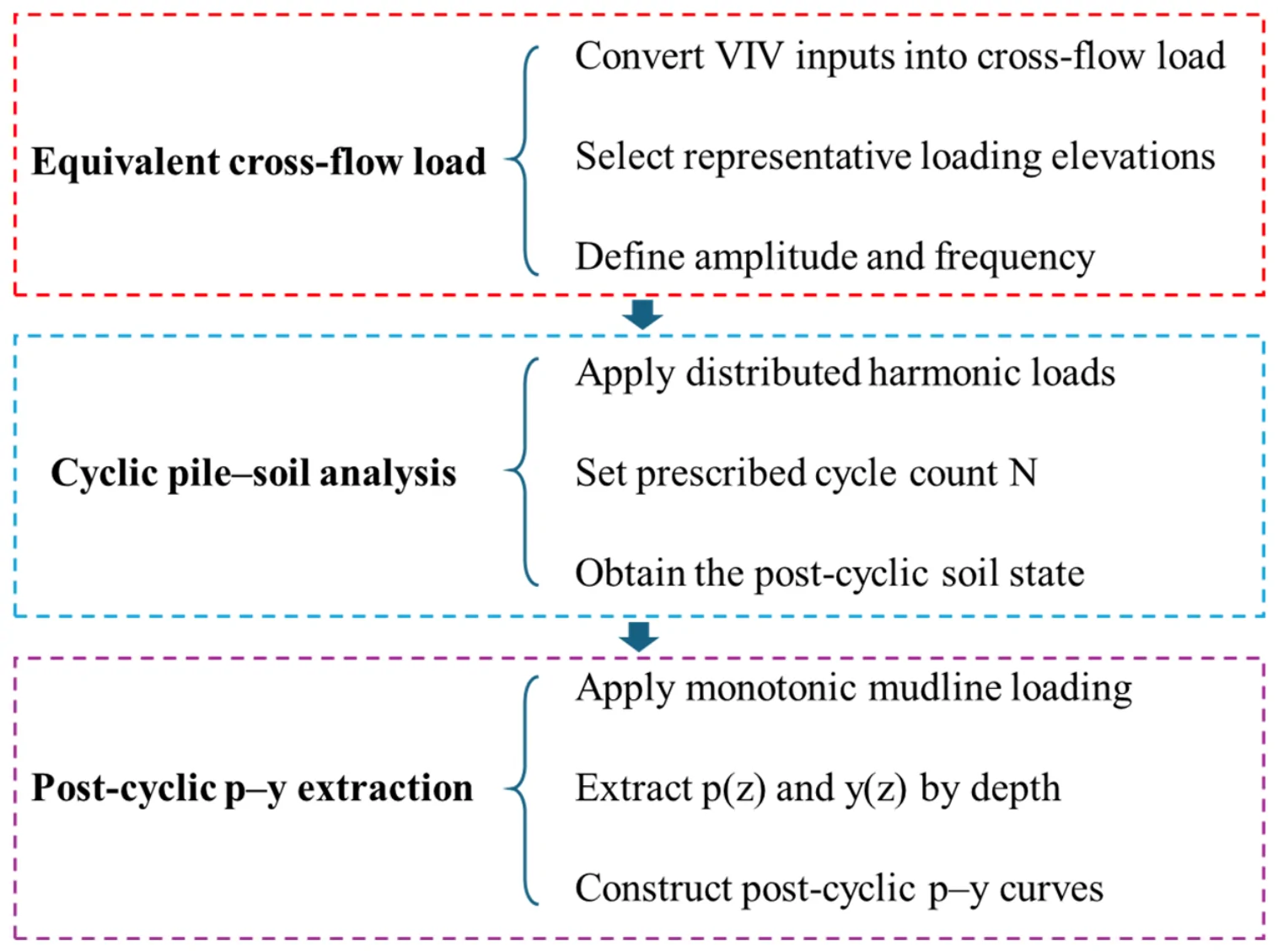
Schematic of the equivalent vortex-induced cross-flow loading and procedure for extracting post-cyclic p–y curves.

The finite-element p–y curves were compared with the sand p–y formulation recommended by the American Petroleum Institute (API) [24], which is expressed as

(2) $$p=A\times p_{u}\tanh\left(\frac{k\times z}{A\times \mathrm{pu}}y\right)$$

(3) $$p_{u}=\min\{(C_{1}z+C_{2}D)\gamma z,C_{3}D\gamma z\}$$

where *p*_u_ is the ultimate soil resistance per unit pile length, *k* is the horizontal soil-reaction coefficient, *z* is the depth below the seabed, The parameter γ in Equation (3) denotes the effective submerged unit weight of sand and is distinct from the saturated unit weight γ_sat_ = 20.8 kN/m^3^ reported in Table 1. Under hydrostatic conditions, γ = γ_sat_ − γw. Using γ_w_ = 9.81 kN/m^3^ gives γ = 10.99 kN/m^3^, approximately 11.0 kN/m^3^. C_1_, C_2_, and C_3_ are coefficients determined by the sand friction angle. For static loading, the loading factor was calculated as

(4) $$A=\left(3.0-0.8\frac{z}{D}\right)\geq 0.9$$

whereas *A* = 0.9 was used for the cyclic-loading condition. The API curves were used as a reference to evaluate the initial lateral stiffness and the cycle-induced degradation of the p–y response obtained from the finite-element model. The overall computational framework is summarized in Figure 3. The equivalent cross-flow loading scheme and the procedure for extracting the post-cyclic p–y curves are illustrated in Figure 3.

## 3. Results

For monopile foundations, small changes in lateral stiffness can influence mudline displacement, rotation, bending demand, and the dynamic characteristics of the supported turbine. The peak in-line drag and cross-flow lift first need to be examined under static loading to identify which component controls the immediate structural response and to establish the initial p–y baseline. The cross-flow lift, however, acts cyclically with vortex shedding; even when its static effect is modest, repeated loading may progressively disturb the surrounding sand and reduce the soil resistance mobilized at a given displacement. It is therefore necessary to distinguish the short-term static response from the cycle-dependent evolution of the pile–soil interaction before assessing whether the conventional API p–y relation remains adequate.

### 3.1. Static Lateral Response and p–y Curves

**Figure 4:**
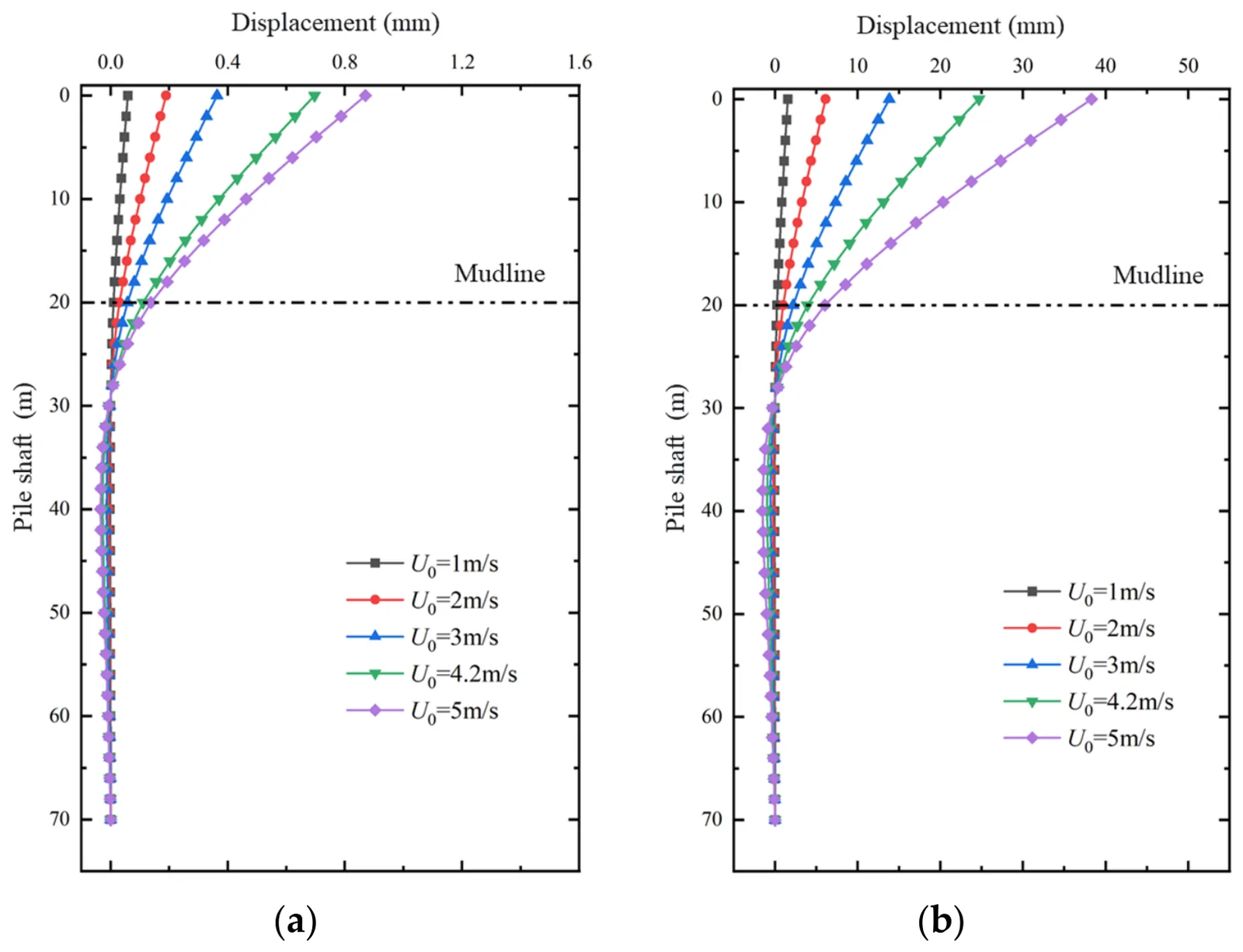
Pile displacement profiles under different current velocities: (**a**) cross-flow loading and (**b**) in-line loading.

**Figure 5:**
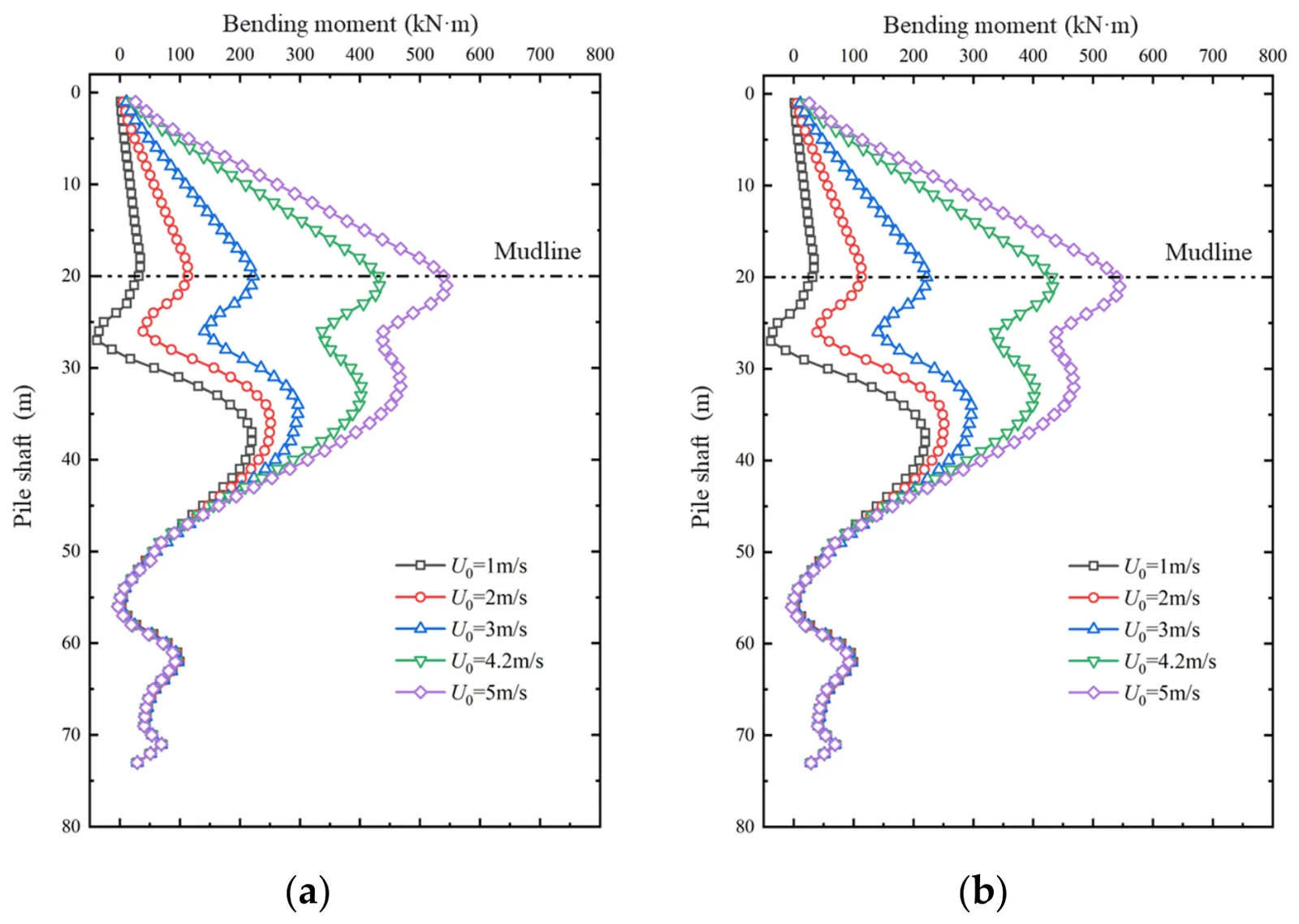
Pile bending-moment profiles under different current velocities: (**a**) cross-flow loading and (**b**) in-line loading.

The static responses of the monopile under the maximum in-line and cross-flow loads are shown in Figure 4 and Figure 5. For both loading directions, the lateral displacement increased with current velocity and decreased rapidly after the pile entered the seabed. Under the static cross-flow loads, the maximum pile-head displacement increased from 0.05 mm at the lowest current velocity to 0.87 mm at the highest current velocity. The displacement was mainly concentrated within the exposed pile segment and the shallow embedded zone, approaching zero at a depth of approximately 27 m along the pile. At the higher load levels, a small displacement reversal occurred in the deeper embedded segment as a result of the restraint provided by the surrounding soil.

The in-line loads produced substantially larger pile displacements because the corresponding current-induced drag forces were much greater than the cross-flow lift forces. The maximum pile-head displacement increased from 0.15 mm to 38.3 mm as the current velocity increased. A more pronounced displacement reversal developed at depths of approximately 30–55 m, with a maximum reverse displacement of about 1.5 mm in the highest-velocity case. The bending-moment profiles exhibited a similar dependence on the loading direction. Under cross-flow loading, the maximum bending moment increased from 34.4 kN·m to 544 kN·m as the current velocity increased from 1 to 5 m/s. In contrast, the bending-moment profiles under in-line loading were smoother, with the moment decreasing gradually toward zero below the depth of approximately 25 m. These results indicate that the in-line current load governs the overall static deformation of the monopile, whereas the smaller cross-flow load provides the basis for the subsequent cyclic pile–soil interaction analysis.

**Figure 6:**
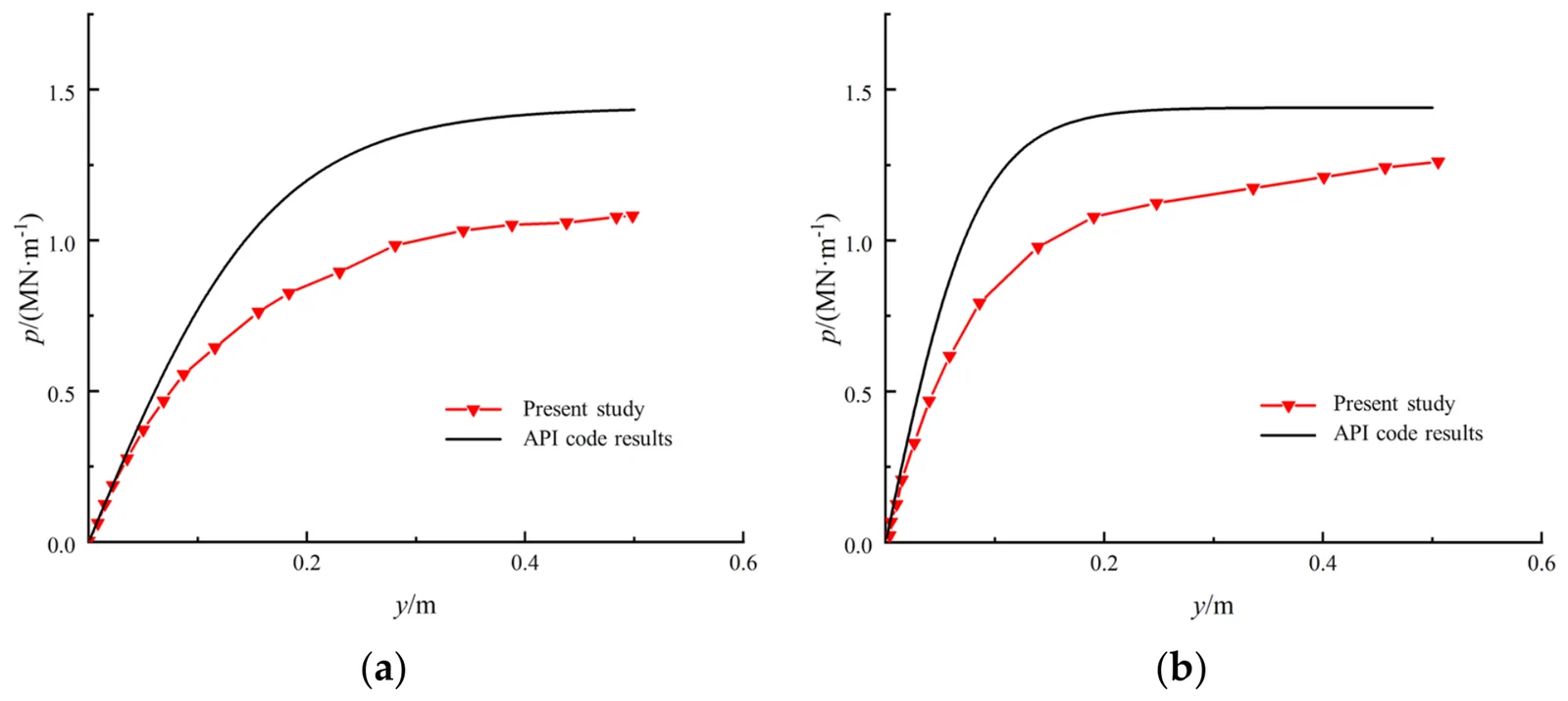
Static p-y curves from the finite-element model and API relation at (**a**) z = 2/D and (**b**) z = D.

The static p–y curves obtained from the finite-element model at depths of *z* = 2/D and *z* = D are compared with the API sand formulation in Figure 6. At both depths, the numerical and API curves exhibit a similar nonlinear increase in soil resistance with lateral displacement. However, the finite-element results remain below the API predictions over the investigated displacement range, with the difference being most evident at lateral displacements between approximately 0.15 and 0.40 m. This discrepancy indicates that the API formulation overestimates the initial soil-reaction modulus for the investigated large-diameter monopile–sand system.

Within the shallow zone of z ≤ D, the numerical soil resistance approached its limiting value only when the lateral displacement exceeded approximately 0.3 m. Therefore, although the API formulation reproduces the general nonlinear trend of the p–y response, it does not adequately capture the initial lateral stiffness predicted by the finite-element model. The static results provide the reference state for evaluating the subsequent changes in p–y behaviour induced by vortex-generated cyclic cross-flow loading.

### 3.2. Cyclic Degradation of the p–y Response

**Figure 7:**
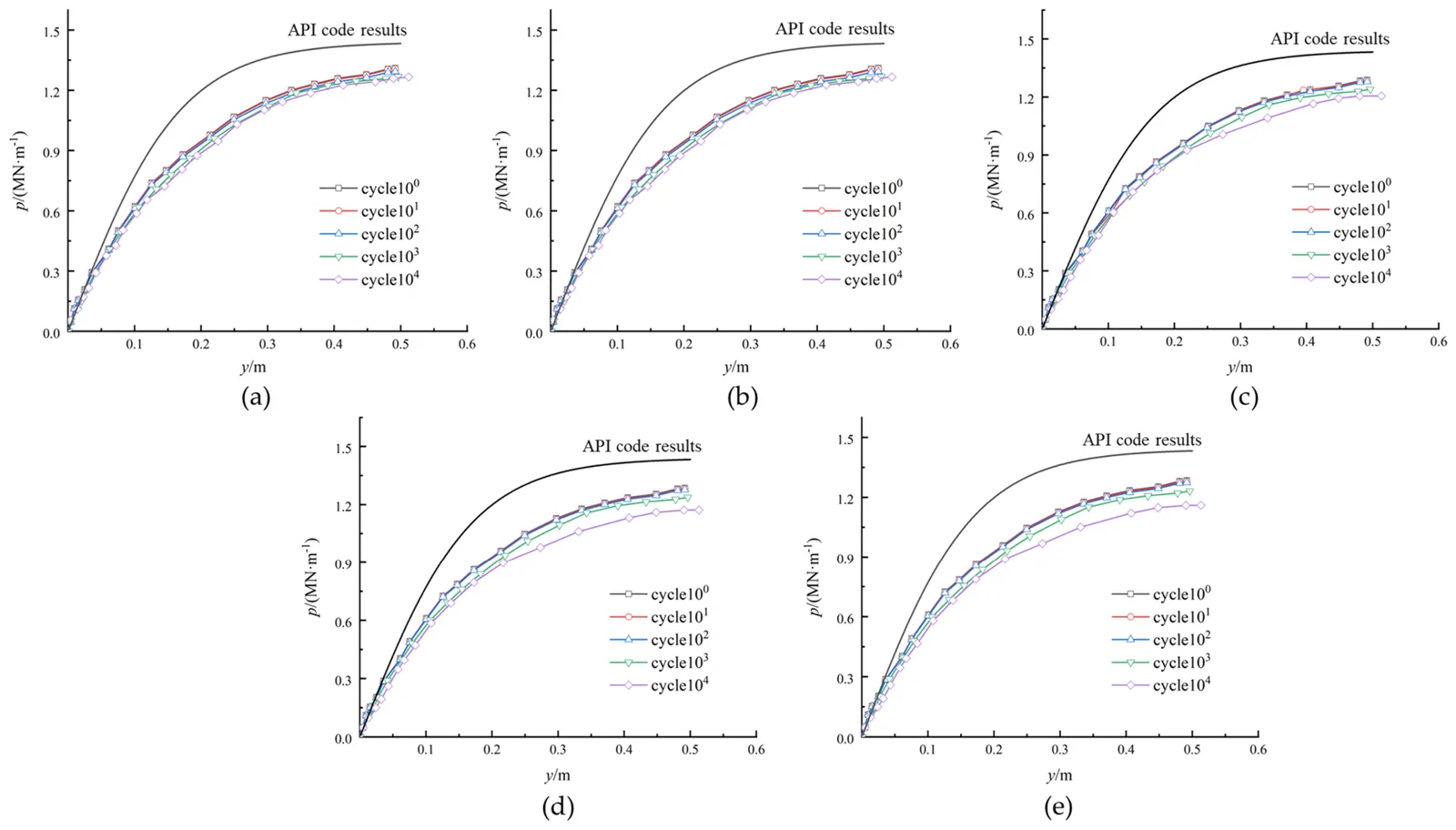
Effect of load cycles on the p-y response at z = D/2 for the five current-velocity cases: (**a**) Case 1; (**b**) Case 2; (**c**) Case 3; (**d**) Case 4; (**e**) Case 5.

The evolution of the p–y response under vortex-induced cross-flow loading was evaluated after 10^0^, 10^1^, 10^2^, 10^3^, and 10^4^ loading cycles. Figure 7 presents the p–y curves at z = D/2 for Cases 1–5. The API curve is included as a reference. For each loading case and depth, the curve obtained after one cycle serves as the reference for evaluating subsequent cyclic changes. At a fixed lateral displacement y, the percentage reduction in mobilized soil resistance is expressed as R_p_(N,y) = [p_n=1_(y) − p_n_(y)]/p_n=1_(y) × 100%, where p_n=1_(y) and p_n_(y) are the soil resistances after one and N loading cycles, respectively. Resistance degradation is quantified by this fixed-displacement comparison, while stiffness degradation is characterized by changes in the initial tangent modulus K.

In all five cases, the finite-element curves shifted progressively downward as the number of cycles increased, indicating a gradual reduction in the lateral resistance of the surrounding sand. The curves remained relatively close at the lower cycle counts, whereas a more evident reduction developed after 10^3^–10^4^ cycles.

The magnitude of degradation also increased with the cyclic-load level. In Case 1, corresponding to the lowest current velocity and cyclic-load amplitude, the change in soil resistance remained small up to 10^3^ cycles. After 10^4^ cycles, the soil resistance at *y* = 0.5 m decreased by approximately 6.0% relative to the initial response. By comparison, Case 5 produced a reduction of approximately 12.5% at the same displacement after 10^4^ cycles. Cases 2–4 exhibited intermediate responses, confirming that the accumulated reduction in soil resistance generally became more pronounced as the cyclic cross-flow load increased.

**Figure 8:**
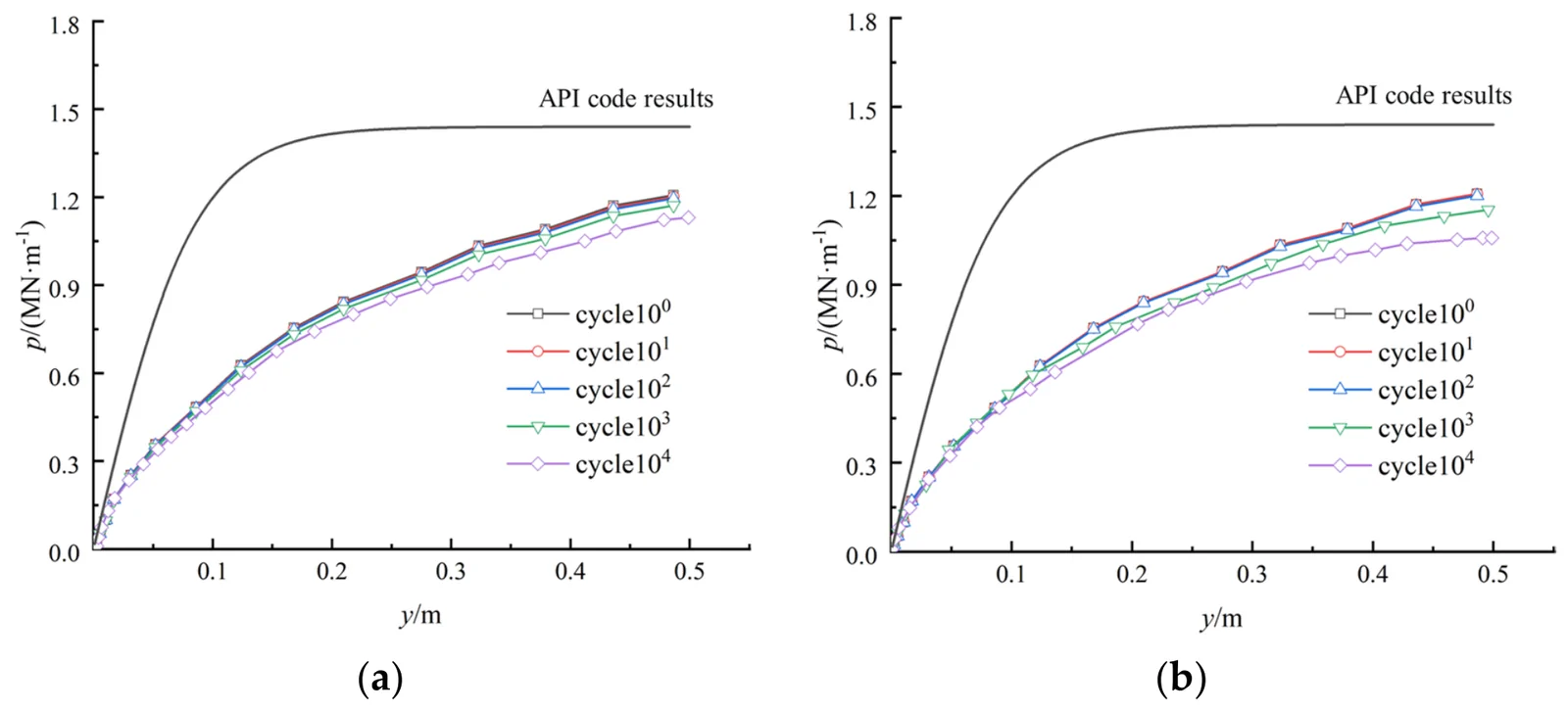
Evolution of post-cyclic p–y curves at z = D after 10^0^, 10^1^, 10^2^, 10^3^ and 10^4^ loading cycles: (**a**) Case 1 (U = 1 m/s), (**b**) Case 5 (U = 5 m/s).

To further examine the cyclic response at a greater depth, Figure 8 compares Cases 1 and 5 at *z* = *D*. In Case 1, a discernible reduction in soil resistance emerged after 10^3^ cycles. After 10^4^ cycles, the maximum reduction reached approximately 6.75% at a lateral displacement of (y = 0.33) m. The higher cyclic-load level in Case 5 produced a clearer downward shift after 10^3^ cycles, and the maximum reduction reached approximately 13.4% at *y* = 0.45 m after 10^4^ cycles. These results demonstrate that the cyclic degradation was not confined to the soil immediately below the seabed but remained evident at a depth equal to one pile diameter.

The influence of cyclic loading was limited at small lateral displacements and became increasingly evident as the soil resistance was more fully mobilized. This behaviour indicates that the degradation of the p–y response depends jointly on the number of cycles, the cyclic-load level, the observation depth, and the magnitude of pile displacement. These changes in mobilized soil resistance represent numerical results for the investigated monopile–soil configuration. The comparison also identifies two limitations of the unmodified API formulation for the investigated monopile–sand system: it overestimates the static soil resistance over part of the displacement range and does not explicitly represent the progressive stiffness reduction caused by repeated vortex-induced loading.

### 3.3. Modified p–y Formulation

The results presented in Section 3.1 and Section 3.2 indicate that two modifications are required to represent the lateral soil response of the investigated monopile–sand system. First, the API formulation overestimates the initial soil-reaction modulus obtained from the finite-element model. Second, its constant cyclic-loading factor does not represent the progressive stiffness reduction associated with the number and amplitude of vortex-induced loading cycles. The proposed modification therefore incorporates a depth-dependent reference modulus and a cycle-dependent degradation coefficient.

**Figure 9:**
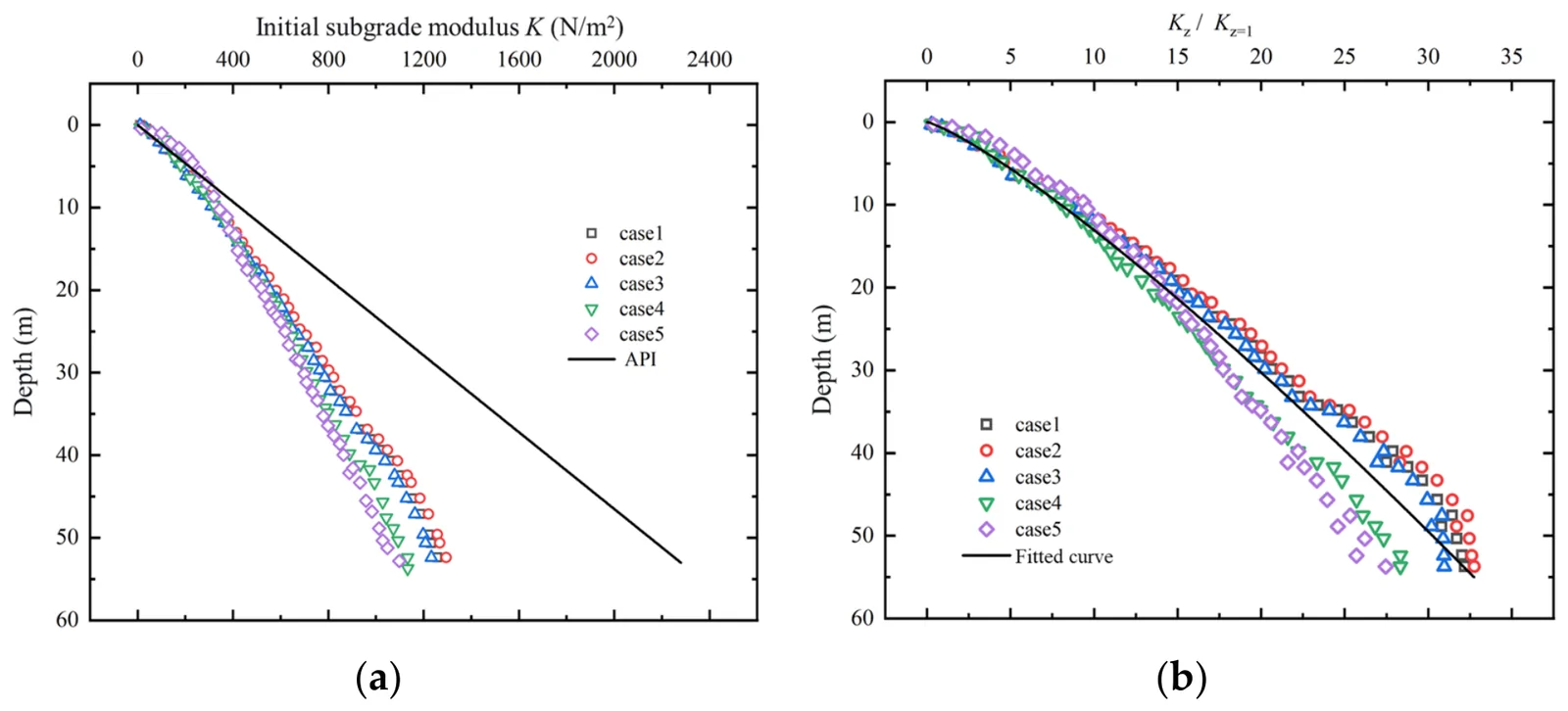
Depth dependence of the initial soil-reaction modulus: (**a**) comparison between the finite-element results and the API formulation; (**b**) normalized modulus and fitted power-law relationship.

In the API formulation, the initial soil-reaction modulus at depth *z* is expressed as

(5) $$K_{\mathrm{API}}=k\times z$$

where *k* is the horizontal soil-reaction coefficient. This expression assumes that the initial modulus increases linearly with depth. The values extracted from the finite-element p–y curves, however, exhibited a nonlinear depth dependence and remained below the API predictions over the investigated depth range, as shown in Figure 9a. To quantify this difference, the depth-dependent modulus was expressed in the general form

(6) $$K_{z}=a\times k\times z_{\mathrm{ref}}{\left(\frac{z}{z_{\mathrm{ref}}}\right)}^{b}$$

where *a* and *b* are fitting parameters. The modulus values were normalized using the value at *z* = 1 m before regression. The resulting relationship, shown in Figure 9b, was

(7) $$K_{z}=1.2k\times z_{\mathrm{ref}}{\left(\frac{z}{z_{\mathrm{ref}}}\right)}^{0.85},z_{\mathrm{ref}}=1\;m$$

where z_ref_ = 1 m is the reference length, and a and b are dimensionless fitting parameters. The dimensionless depth is defined as z/z_ref_, and the corresponding stiffness scale is kz_ref_. Introducing this reference length ensures dimensional consistency and preserves the numerical values of the original expression when depth is expressed in metres.

A degradation coefficient, *λ*, was subsequently introduced to account for the reduction in the initial soil-reaction modulus under repeated cross-flow loading:

(8) $$\lambda =\frac{K_{\mathrm{ini}}^{N}}{K_{z}}=N^{-r}$$

where *K_ini_^N^* is the initial soil-reaction modulus after *N* loading cycles, *K_z_* is the corrected reference modulus, and *r* is the cyclic weakening factor. Taking the logarithm of both sides gives

(9) $$\log\lambda_{N}=-r\log N$$

**Figure 10:**
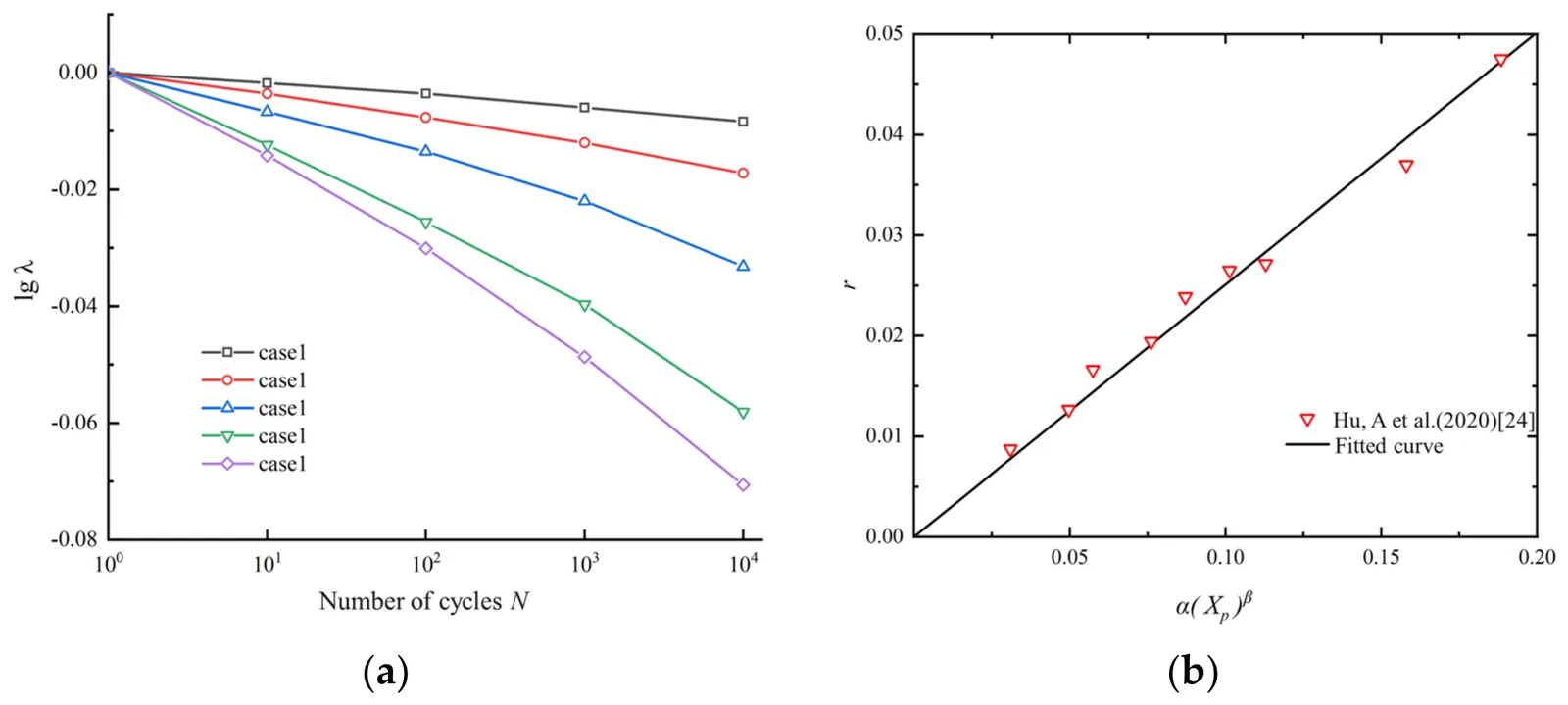
Cyclic-stiffness degradation: (**a**) relation between cycle count and the degradation coefficient at *z* = *D*/2, (**b**) fitted relation between r and *α*(*X_p_*)*^β^*.

Accordingly, the ordinate in Figure 10a is log*λ_N_*, while the number of cycles, *N*, is plotted on a logarithmic abscissa. The approximately linear relationships between log*λ_N_* and log*N* support the adopted power-law degradation model, and the negative slope of each fitted line corresponds to −*r*. The power law is an empirical representation of cyclic changes in the initial soil-reaction modulus. The exponent r measures the rate of stiffness reduction per logarithmic increase in cycle number. As the cyclic-load level increased, the absolute value of the fitted slope generally increased, indicating a more pronounced degradation of the initial soil-reaction modulus. Cases 1 and 2 exhibited relatively limited degradation, whereas Cases 3–5 showed progressively greater reductions.

The power-law form of λ_N_ follows the stiffness-degradation framework proposed by Huurman [23]:

(10) $$\frac{E_{\mathrm{sN}}}{E_{s1}}=\frac{\varepsilon_{p,1}^{a}}{\varepsilon_{p,N}^{a}}=N^{-\alpha {\left(X\right)}^{\beta }}$$

(11) $$X=\frac{\sigma_{1,c}}{\sigma_{1,\mathrm{sf}}}$$

where *E_s_*_,1_ and *E_s,N_* are the sand elastic moduli after the first and *N*th cycles, *ε^a^_p,_*_1_ and *ε^a^_p,N_* are the corresponding axial plastic strains, and *X* is the cyclic stress ratio. The parameters *α* and *β* depend on the sand and may be obtained from triaxial tests; the values used in the adopted relation are summarized in Table 4.

For application to laterally loaded piles, Hu et al. [24] represented the cyclic loading level using the mobilized soil-resistance ratio.

(12) $$X_{p}=\frac{p_{1}}{p_{u}}$$

For each loading case and depth, p_1_ denotes the magnitude of the local soil reaction at the peak load in the first cycle, and p_u_ is evaluated from Equation (3) at the same depth. Their ratio, X_p_, characterizes the mobilized loading level. For comparison, the empirical relationship associated with the reference data in Figure 10b is expressed as

(13) $$r=0.251\times \alpha {\left(X_{p}\right)}^{\beta }$$

where *α* and *β* are parameters of the reference relationship. Equation (13) provides a framework for interpreting the loading dependence of the weakening factors obtained in this study by relating cyclic weakening to the mobilized soil-reaction ratio [25]. Within this framework, Figure 10a provides the case-specific calibration of r from the numerical stiffness evolution, while Figure 10b illustrates the empirical basis for interpreting its dependence on loading level. The values of r adopted for the present cases are obtained from the negative slopes of the fitted relationships between logλ_N_ and logN.

Depth dependence is represented through the reference modulus K_z_, while r is held fixed for each evaluated loading case and depth when constructing the post-cyclic p–y curve. This simplification describes changes in the initial stiffness without implying that the physical degradation process is independent of depth or displacement.

From the definition of the degradation coefficient, the effective initial soil-reaction modulus after *N* loading cycles is given by

(14) $$K_{\mathrm{ini}}^{N}=\lambda_{N}K_{z}$$

To incorporate both the depth-dependent reference modulus and the cycle-induced stiffness degradation, *K_ini_^N^* was substituted for the original *kz* term in the API p–y formulation. The resulting modified relationship is

(15) $$p_{N}=Ap_{u}\tanh(\frac{\lambda_{N}K_{z}}{Ap_{u}}y)$$

Substituting *K_z_* = 1.2 *kz*^0.85^ into the above equation gives

(16) $$p_{N}=Ap_{u}\tanh(\frac{1.2\lambda_{N}kz_{\mathrm{ref}}}{Ap_{u}}{\left(\frac{z}{z_{\mathrm{ref}}}\right)}^{0.85}y)$$

where *p_N_* is the lateral soil resistance after *N* loading cycles. For the reference condition without cyclic degradation, *λ_N_* = 1. Following cyclic loading, the degradation coefficient is calculated as

(17) $$\lambda_{N}=N^{-r}$$

where *r* is determined from the mobilized soil-resistance ratio. This substitution incorporates the depth-dependent correction and cycle-dependent stiffness reduction into the original functional form of the API p–y model. The resulting formulation combines the depth-dependent stiffness correction and the cycle-dependent degradation relationship, calibrated against the finite-element results, to describe the post-cyclic p–y response of the investigated monopile–soil system.

The proposed formulation modifies the initial stiffness while retaining the API resistance parameter p_u_. For positive displacement, the initial slope is λ_N_K_z_ and the asymptotic resistance is Ap_u_. Consequently, a lower stiffness produces a lower mobilized resistance at a given finite displacement without requiring a change in the asymptotic resistance. The formulation describes the stiffness-controlled response within the investigated displacement range for one pile geometry, one soil parameter set, and five harmonic loading cases, with cyclic p–y comparisons focused on two shallow depths and up to 10^4^ cycles. Independent validation and assessment of prediction uncertainty, extension to service-life cycling and irregular, multidirectional loading, and a separate treatment of cyclic changes in ultimate resistance are planned for future research.

## 4. Discussion

The numerical and API p–y responses differ in both reference stiffness and cyclic evolution. The depth-dependent correction addresses the nonlinear variation in the initial soil-reaction modulus, while the cyclic coefficient describes its subsequent change with loading history. Separating these effects avoids attributing the entire discrepancy to cyclic loading. Within the modified formulation, reduced stiffness increases the displacement required to mobilize a specified resistance. Consequently, lower resistance at a given displacement can be represented without changing the prescribed asymptotic resistance.

The downward shifts in the post-cyclic p–y curves at both examined depths indicate that the cyclic influence extends below the soil immediately adjacent to the seabed. These shifts characterize changes in the modelled pile–soil response. Since the Mohr–Coulomb parameters remain fixed, they do not directly establish an intrinsic stiffness-degradation law for sand. The fitted weakening factor instead summarizes the numerical stiffness evolution under each investigated loading condition.

Differences among the five cases reflect the combined effects of load amplitude and application elevation. A larger amplitude increases the lateral force, while a higher application point increases its moment about the seabed. The stronger degradation in the higher-velocity cases therefore reflects the corresponding amplitude–elevation combinations and cannot be attributed to current velocity alone. Independent application of these cases permits comparison of representative loading conditions, although it does not reproduce a simultaneous, spatially varying VIV load field.

Existing approaches describe cyclic monopile response through modified p–y curves, cyclic overlays, or reaction-ratio relationships [4,11,21]. The present framework complements these approaches by linking equivalent cross-flow loading to the post-cyclic response while separating reference stiffness from cyclic correction. Its engineering relevance concerns the soil support mobilized under serviceability loads, for which stiffness changes can influence pile displacement and rotation. Supported by monotonic validation of the reference model, the formulation describes the investigated pile–soil response under harmonic loading within the examined cycle range, while independent cyclic validation would strengthen its broader applicability.

## 5. Conclusions

This study used a validated three-dimensional finite-element model to investigate the static and cyclic lateral response of a large-diameter offshore wind monopile in sand under current-induced loading. Particular attention was given to the evolution and modification of p–y curves under repeated vortex-induced cross-flow loading. The principal conclusions are as follows:

1. The validated model showed that the in-line current force governed the short-term static response of the monopile, producing substantially greater displacement and bending moment than the peak cross-flow lift. Although the static effect of the cross-flow load was limited, its repeated action provided the primary mechanism for the progressive change in pile–soil interaction.
2. The API formulation reproduced the general nonlinear shape of the *p–y* response but overestimated the initial soil-reaction modulus for the investigated monopile–sand system. Repeated cross-flow loading progressively reduced the lateral soil resistance, and the degradation increased with the number of cycles, cyclic-load level, and mobilized displacement. After 10^4^ cycles, the reduction in soil resistance reached approximately 6.0–12.5% at *z* = *D*/2 and 6.75–13.4% at *z* = *D* across the representative low- and high-load cases.
3. A modified p–y approach was developed by combining a nonlinear depth-dependent reference modulus with a cycle-dependent stiffness-degradation coefficient. The proposed approach retains the functional form of the conventional API model while representing both the lower initial stiffness predicted by the finite-element analysis and its progressive reduction under vortex-induced cyclic loading.

Overall, this study links vortex-induced cross-flow loading with the long-term degradation of monopile p–y response and provides an engineering-oriented framework for incorporating this effect into lateral-response analysis. Its applicability is bounded by the simplified harmonic VIV loading, the adopted Mohr–Coulomb model, and the investigated range of up to 10^4^ cycles. Future work should independently validate the formulation and extend its calibration to broader pile–soil conditions, irregular loading, and service-life cycle ranges.

## Figures and Tables

**Table 1:** Parameters of the validation and engineering-scale pile-soil models.

Component	Parameter	Validation Model	Engineering Model
Pile	Total length (m)	65	73
	Embedded length (m)	50	53
	Outer diameter (m)	2.5	4.5
	Wall thickness (m)	0.045	0.05
	Elastic modulus (GPa)	68.9	210
	Poisson’s ratio	0.30	0.30
Soil	Unit weight (kN/m^3^)	8.45 (effective)	20.8 (saturated)
	Cohesion (kPa)	0.05	5
	Friction angle (°)	35	39
	Dilation angle (°)	17.5	9
	Elastic modulus (MPa)	-	56
	Poisson’s ratio	0.21	0.30
Interface	Friction coefficient	-	0.35

**Table 2:** Static lateral-load cases for the engineering-scale monopile.

Case	Velocity (m/s)	Cross-Flow Load (kN)	In-Line Load (kN)
1	1.0	2.0	53
2	2.0	6.5	211
3	3.0	12.5	476
4	4.0	24.0	850
5	5.0	30.0	1320

**Table 3:** Cyclic cross-flow load cases and application elevations.

Case	Velocity (m/s)	Peak Load (kN)	Shedding Frequency (Hz)	Elevation (m)	*B_L_*_,_*_a_* (m)
1	1.0	1.50	0.13	4	0.668003
2	2.0	4.05	0.15	8	0.450902
3	3.0	9.11	0.13	12	0.450778
4	4.0	14.40	0.12	16	0.350746
5	5.0	19.69	0.12	20	0.400802

**Table 4:** Sand stiffness-degradation parameters used in the cyclic model.

Sand	α	β
Sand A	18.6	0.48
Sand B	10.1	0.60
Sand C	12.6	0.55
Sand D	6.3	0.72
Sand E	7.4	0.62
Sand F	5.4	0.69
Sand G	8.2	0.67
Crusher sand	5.7	0.55

## Data Availability

The original contributions presented in this study are included in the article. Further inquiries can be directed to the corresponding author.
